# Template-Mediated Synthesis of Hierarchically Porous Metal–Organic Frameworks for Efficient CO_2_/N_2_ Separation

**DOI:** 10.3390/ma15155292

**Published:** 2022-07-31

**Authors:** Tianjie Qiu, Song Gao, Yanchun Fu, Dong Xu, Dekai Kong

**Affiliations:** 1 Beijing Key Laboratory for Theory and Technology of Advanced Battery Materials, School of Materials Science and Engineering, Peking University, Beijing 100871, China; qtjie@pku.edu.cn; 2 CHN Energy New Energy Technology Research Institute Co., Ltd., Beijing 102211, China; 12050383@chnenergy.com.cn (Y.F.); dong.xu@chnenergy.com.cn (D.X.); 3 State Key Laboratory of Heavy Oil Processing, China University of Petroleum-Beijing, Beijing 102249, China; kongdekai6@163.com

**Keywords:** CO_2_ separation, metal–organic frameworks, hierarchically porous structures, template-mediated synthesis

## Abstract

Carbon dioxide (CO_2_) is generally unavoidable during the production of fuel gases such as hydrogen (H_2_) from steam reformation and syngas composed of carbon monoxide (CO) and hydrogen (H_2_). Efficient separation of CO_2_ from these gases is highly important to improve the energetic utilization efficiency and prevent poisoning during specific applications. Metal–organic frameworks (MOFs), featuring ordered porous frameworks, high surface areas and tunable pore structures, are emerging porous materials utilized as solid adsorbents for efficient CO_2_ capture and separation. Furthermore, the construction of hierarchical MOFs with micropores and mesopores could further promote the dynamic separation processes, accelerating the diffusion of gas flow and exposing more adsorptive pore surface. Herein, we report a simple, efficient, one-pot template-mediated strategy to fabricate a hierarchically porous CuBTC (CuBTC-Water, BTC = 1,3,5-benzenetricarboxylate) for CO_2_ separation, which demonstrates abundant mesopores and the superb dynamic separation ability of CO_2_/N_2_. Therefore, CuBTC-Water demonstrated a CO_2_ uptake of 180.529 cm^3^ g^−1^ at 273 K and 1 bar, and 94.147 cm^3^ g^−1^ at 298 K and 1 bar, with selectivity for CO_2_/N_2_ mixtures as high as 56.547 at 273 K, much higher than microporous CuBTC. This work opens up a novel avenue to facilely fabricate hierarchically porous MOFs through one-pot synthesis for efficient dynamic CO_2_ separation.

## 1. Introduction

To mitigate the increasingly severe issues of global warming and climate change, extensive concerns have been concentrated on developing effective separation/capture systems for selective removal of CO_2_ from gaseous mixtures. Selective separation of CO_2_ is also paramount for the purification of valuable fuel gases such as H_2_ and CH_4_ [1,2]. Therefore, it is urgent that a large-capacity and high-selectivity adsorbent system with high feasibility of preparation and low energy consumption for CO_2_ separation is developed. Recently, by virtue of the highly tunable pore structure, abundant accessible adsorptive sites and well-defined crystalline structure, metal–organic frameworks (MOFs) have shown great potential for gas separation and capture through chemisorption/physisorption [3,4,5]. Compared with traditional porous materials such as porous carbon and molecular sieves, the highly tunable MOF structures can be subtly functionalized with functional groups, open metal sites and favorable pore shapes to judiciously optimize the microscopic pore environments and improve CO_2_ adsorption capacity and selectivity [6,7]. As a typical example, Mg-MOF-74 materials with abundant open metal sites have been reported to exhibit high efficiency for CO_2_ separation from CO_2_/CH_4_ mixtures [8]. In addition, grafting basic amine as functional groups in the pore surface via pre-modification with amine-functionalized ligands or post-introduction of amine molecules could also enhance the adsorptivity and selectivity of MOFs through Lewis acid–base interaction between MOFs and CO_2_ molecules [7,9,10]. However, the success of microporous MOFs under equilibrium conditions in adsorbing pure CO_2_ cannot be guaranteed when exposed to the gas mixtures under realistic dynamic conditions because of the high sorption and mass transfer limitations of microporous MOFs [9,11,12]. Trade-offs between permeability and selectivity of MOF adsorbents should be carefully considered for realistic dynamic CO_2_ separation, which is still a huge change for realistic large-scale separation [13].

Engineering the structure of MOFs into hierarchical nanoarchitecture has recently drawn increasing concerns, such as the creation of larger pore sizes or hollow structures to enhance the dynamic capacity and selectivity [14,15,16]. It has been discovered that the hierarchical porosity from the micro- to meso-scale could fully make use of intrinsic pores of MOFs with higher exposure to the gas molecule, which can also effectively accelerate the mass diffusion/permeation and avoid blocking of the micropores. Larger-size meso- and macroporous channels also provide enough space to load functional groups to further enhance the separation efficiency, which also presents great potential in many applications, such as heterocatalysis and drug delivery [17,18,19,20]. For the synthesis of hierarchical MOFs, increasing the length of linkers is a direct way to construct intrinsic hierarchical pores while the total routes involving fabrication of the specific linkers and topological MOFs are always a time/energy-consuming process [21,22]. In addition, researchers have also adopted post-modulation and template-directed methods to create hierarchical MOFs [23]. For example, Jiang et al. developed a general method using monocarboxylic acid as the modulator to fabricate a series of hierarchical MOFs [24]. Chen et al. employed polystyrene (PS) nanosphere monoliths as a hard template to synthesize an ordered macro–microporous ZIF-8 single crystal, which opened up an avenue for the design of large-size hierarchically porous MOFs with single-crystal structures [25]. However, developing a simple and one-pot method to directly fabricate hierarchically porous MOFs with both micro- and mesopores still remains a challenge.

Generally, most reported hierarchical CuBTC MOFs were composed of stacked small MOF particles using dissolvable Cu^2+^ metal salts rather than inherent hierarchical pores. In our work, we developed a facile one-pot self-sacrifice template strategy to fabricate a CuBTC-Water MOF based on a Cu_2_O precursor as the sole Cu^+^ metal source, which led to many defects in the framework and contributed to the hierarchical porous structures. This strategy demonstrated quite a simple preparation process to obtain the hierarchical CuBTC MOF without further post-treatment or templates, unlike conventional methods [26,27,28]. The hierarchically porous CuBTC-Water demonstrated a high CO_2_ adsorption ability under static adsorption–desorption conditions. Such hierarchically porous CuBTC-Water also exhibited more efficient dynamic separation behavior toward CO_2_/N_2_ and CO_2_/H_2_ than microporous CuBTC under continuous gas flow conditions, owing to its enhanced adsorption kinetics through the shortened diffusion pathway and highly exposed adsorptive surface. We hope this work can guide further study of hierarchically porous MOFs in service of industrial high-efficiency CO_2_ separation.

## 2. Experimental Section

### 2.1. Materials and Characterizations

All reagents and solvents were used directly as received from commercial sources without further purification. Thermo gravimetric analysis (TGA) was conducted using an SDT Q600 analyzer (TA Instruments, New Castle, DE, USA) under a continuous nitrogen flow (100 mL min^−1^) at a heating rate of 10 °C min^−1^.Powder X-ray diffraction (PXRD) patterns for the materials were collected with a (Rigaku Corporation SmartLab 9 kW diffractometer, Japan) at 45 kV and 200 mA with Cu Kα radiation (λ = 1.5406 Å). Scanning electron microscopy (SEM) images were recorded through a (Hitachi SU-8200 electron microscope, Japan). Fourier transform infrared spectroscopy (FT-IR) spectra were recorded by a (Bruker Tensor 27 Fourier transform infrared spectrometer, Germany) from 400 to 4000 cm^−1^ (KBr pellets). Chemical states were analyzed using X-ray photoelectron spectroscopy (XPS, Thermo Scientific Nexsa, Czech Republic).

### 2.2. Synthesis of CuBTCMOF

The synthesis of traditional CuBTC MOF was conducted using a solvothermal method. In detail, 8.4 g of H_3_BTC was dissolved in 240 mL of ethanol, and 17.5 g of Cu(NO_3_)_2_ 3H_2_O was dissolved in 240 mL of deionized water. Then, the ethanol solution with the organic ligand and aqueous solution with the metal source were mixed to form a homogeneous solution; thereafter, the mixed solution was separated into six Teflon reactors at 120 °C for 10 h. Finally, the produced samples were collected and dried in a vacuum oven at 150 °C for activation.

### 2.3. Synthesis of CuBTC-DMF

Firstly, 0.234 g of commercialized Cu_2_O was weighed and put in a 100 mL clean beaker. Then, 0.4 g of benzenetricarboxylic acid (H_3_BTC) was dissolved in 60 mL of water–ethanol–DMF solution with the volume ratio of 1:1:1. Then, the solution was added into the beaker with Cu_2_O and continuously stirred at room temperature for 24 h. Finally, the blue product was obtained after centrifugation and washed with ethanol to remove the unreacted ligands. The CuBTC-DMF was activated in a vacuum oven at 150 °C for 12 h.

### 2.4. Synthesis of CuBTC-Water

The synthetic method for CuBTC-Water is similar to the synthesis of CuBTC-DMF. 0.4 g of H_3_BTC was dissolved in 60 mL of water–ethanol solution with the volume ratio of 1:1, and 0.234 g of Cu_2_O was mixed with the solution. After stirring at room temperature for 24 h, the blue product was obtained. Finally, the blue product was collected after centrifugation and washed with ethanol. The CuBTC-Water was activated in a vacuum oven at 150 °C for 12 h.

### 2.5. Gas Adsorption Measurements

All the gases (N_2_, CO_2_, H_2_) were of high purity (99.999%). Nitrogen adsorption isotherms at 77 K were performed with a Quantachrome Autosorb-IQ (Quantachrome Instruments, Boynton Beach, FL, USA) gas adsorption analyzer in a liquid nitrogen bath. The CO_2_, N_2_ and H_2_ sorption isotherms were measured at 273 K and 298 K using a circulating glycol–H_2_O bath. All adsorbent materials were activated at 150 °C for 12 h under vacuum conditions. The breakthrough experiments were performed on homemade instruments to simulate the actual mixture gas (20% CO_2_, 20% N_2_ and 60% He) separation to evaluate the adsorbents’ dynamic CO_2_/N_2_ separation ability. Figure S1 shows the home-built and self-designed equipment used for testing the samples’ dynamic gas adsorption and separation performance. The entire equipment comprised air intake, back pressure, mixed sample injection, mass spectrometry and exhaust gas processing. The blue background on the left side of the figure represents the three-way air inlet, the pressure reducing valve and the corresponding mass flow controller; the green background shown in the middle represents the back pressure adjustment pipeline; the yellow background shown in the upper right is the four-way valve, which can be used to switch the gas to enter the adsorption bed or blank bypass; and the white box corresponds to the sample adsorption bed in the circulating temperature-controlled bath, the pressure sensor and the back pressure regulating valve. The final gas pipeline passed through the check valve (marked on the purple background), passing through the mass spectrometer via the capillary pipeline and reaching the computer for the final recording. The mass spectrometer used was a quadrupole mass spectrometer manufactured by PFEIFFER VACUUM, and the instrument model was PrismaPlus QMG220 (Pfeiffer, Germany).

The calculation for Ideal Adsorbed Solution Theory (IAST) selectivity predominantly employs the adsorption isotherms of the two gases at the same temperature and conducts simulation calculations based on the ideal solution adsorption theory. A single Langmuir–Freundlich model was applied to fit the N_2_ with a lower adsorption capacity (as shown in Formula (1)), and a double Langmuir–Freundlich model was used for the CO_2_ with a higher adsorption capacity (as shown in Formula (2)). Subsequently, the IAST theoretical model was employed to simulate the separation selectivity under a certain mixture ratio, and the real ratio of the two gases in this paper was set as the reference (CO_2_:N_2_ = 15:85 (*v*/*v*)).

(1) $$V=\frac{V_{1}\cdot K_{1}\cdot p^{n_{1}}}{{1+K}_{1}\cdot p^{n_{1}}}$$

(2) $$V=\frac{V_{1}\cdot K_{1}\cdot p^{n_{1}}}{{1+K}_{1}\cdot p^{n_{1}}}+\frac{V_{2}\cdot K_{2}\cdot p^{n_{2}}}{{1+K}_{2}\cdot p^{n_{2}}}$$

*v* corresponds to the adsorption capacity per unit mass (cm^3^ g^−1^); *V*_1_ and *V*_2_ represent the saturated adsorption capacity (cm^3^ g^−1^) at two different sites; *K*_1_ and *K*_2_ (kPa^−1^) represent the affinity coefficient (the larger the value of *K*_1_ and *K*_2_, the stronger the interaction between the adsorbate and the adsorbent); *n*_1_ and *n*_2_ represent the deviation value from the ideal homogenous surface (the value of *n*_1_ and *n*_2_ is greater than or equal to 1; the larger the value, the weaker the interaction between the adsorbate and the adsorbent). The selection rules of the adsorption model are based on the nonlinearity of the adsorption isotherm and assume that only one layer of molecules is absorbed between the adsorbent and the adsorbate. Therefore, the physical adsorption model, i.e., the Freundlich model, and the chemical adsorption model, i.e., the Langmuir model, are employed. The adsorption process may simultaneously have physical adsorption and chemical adsorption. Hence, the two models shall be used at the same time. The Langmuir model will be used when *n*_1_ and *n*_2_ equal 1, whereas the Freundlich model is applicable when *p* is at a low level. Since the test process did not reach the high-pressure conditions, the test process complied with a physical adsorption process.

## 3. Results and Discussion

**Scheme 1:**
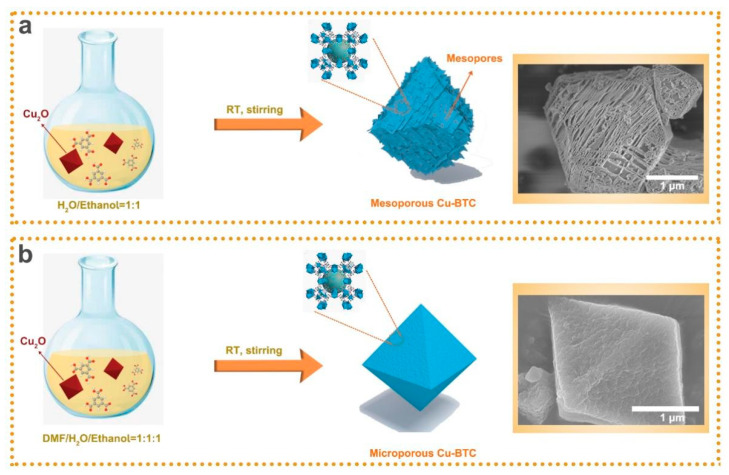
Schematic illustration of the synthesis of hierarchically porous CuBTC-Water (**a**) and microporous CuBTC-DMF (**b**).

As shown in Scheme 1a, the hierarchically porous CuBTC MOF (denoted as CuBTC-Water, where H_3_BTC = 1,3,5-benzentricaboxylic acid) could be synthesized by a facile stirring process at room temperature (RT). The sacrificed solid Cu_2_O was employed as the metal source and could be transformed to the hierarchically porous CuBTC-Water in a water–ethanol solution (volume ratio of 1:1) with organic ligand H_3_BTC dissolved. After continuous stirring, a hierarchically porous CuBTC-Water was finally obtained. Interestingly, the synthesized hierarchical CuBTC-Water displayed an octahedron shape with porous nanosheet-like assembled morphology, which possessed abundant mesopores on its surface (Scheme 1a).

**Figure 1:**
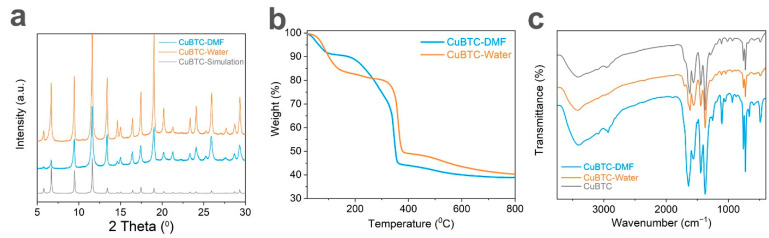
(**a**) PXRD patterns, (**b**) TGA and (**c**) FTIR spectrum of CuBTC-DMF and CuBTC-Water.

From the powder X-ray diffraction (PXRD) pattern (Figure 1a), the formed hierarchically porous CuBTC-Water could match the simulated CuBTC crystal structure well, revealing full conversion of Cu precursors. Unexpectedly, we found that the solvent played an important role in the formation of the hierarchically porous structure. When using water–ethanol/–imethylformamide (DMF) with the volume ratio of 1:1:1 as the solvent, the final product possessed normal octahedron-shape CuBTC morphology with a relatively intact surface, which was quite different from the synthesis without DMF as a solvent (Scheme 1b).

Nevertheless, both CuBTC-Water and CuBTC-DMF possessed the same PXRD patterns with the simulated CuBTC (Figure 1a), which indicated that the difference in solvent would not destroy the framework of the MOF. Figure S2 shows the PXRD patterns of simulated CuBTC, enlarged 6 times, in the 2Ɵ > 13° range, which shows excellent matches. The water stability test involved using a special gas-matched exchange reaction kettle as the test environment container. The CuBTC-Water was placed in the column tank, 5 mL of deionized water was put in the bottom of the reaction kettle, and then the reaction kettle was placed in the oven at 110 °C and maintained for 1 day. After the reactor cooled to room temperature naturally, the CuBTC-Water was removed for subsequent XRD characterization testing. CuBTC-Water still maintained a complete crystal structure, which proved that the material had good water stability (Figure S3). Thermogravimetric analysis (TGA) was also performed to investigate the thermal stability and influence of solvents for the synthesis of these two MOFs. As shown in Figure 1b, both CuBTC-Water and CuBTC-DMF showed sharp weight loss above 350 °C, ascribed to the collapse of MOF frameworks at high temperatures. In addition, the CuBTC-DMF had an obvious mild weight loss at 200–350 °C, indicating the desorption of strongly adsorbed DMF molecules in the pore of CuBTC-DMF during heating. At the same time, CuBTC-Water only displayed a smooth stage at this temperature region, suggesting a clean pore environment. The thermal analysis indicated the stability of such CuBTC-Water was maintained when the hierarchical pores were introduced. CuBTC-Water demonstrated a cleaner pore environment than CuBTC-DMF without the existence of DMF, which is better for the adsorption of CO_2_ molecules. Fourier transform infrared (FTIR) spectroscopy was also used to characterize the crystalline structure of CuBTC-Water and CuBTC-DMF. As shown in Figure 1c, the FTIR spectra of CuBTC-Water were in accordance with those of CuBTC-DMF and pure as-prepared CuBTC, indicating the high-purity HKUST-1 phase after the introduction of hierarchical pores. Unlike the conventional thermal methods, this method provides an energy-efficient route for preparing the hierarchically porous CuBTC-Water at room temperature without any post-treatments to obtain mesopores.

**Figure 2:**
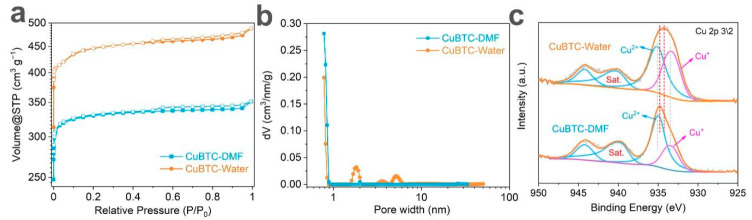
(**a**) N_2_ adsorption and desorption isotherms of CuBTC-DMF and CuBTC-Water at 77 K and (**b**) corresponding pore distributions calculated using NLDFT method. It should be noted that only micropores were observed for the CuBTC-DMF. In contrast, both mesopores and micropores coexisted in the CuBTC-Water. (**c**) High-resolution XPS spectra of CuBTC-DMF and CuBTC-Water.

N_2_ physisorption analysis was then conducted to identify the pore characteristics of CuBTC-Water. As shown in Figure 2a, CuBTC-Water showed a much higher N_2_ uptake at the low-pressure region than CuBTC-DMF, indicating the presence of a large number of micropores and a higher Brunauer–Emmett–Teller (BET) surface area of CuBTC-Water. Figures S4 and S5 show the BET fitting plot of CuBTC-DMF and CuBTC-Water for calculation of their BET specific surface areas. Furthermore, CuBTC-Water showed obvious hysteresis loops at high relative pressure in the N_2_ adsorption–desorption isotherms, which indicated the existence of mesopores. The pore size distribution diagram and SEM image (Scheme 1a) also proved the existence of abundant mesopores as well as microporous structures in CuBTC-Water (Figure 2b). In contrast, CuBTC-DMF merely showed abundant micropores, as shown in Figure 2b and Figure S6. Hence, it can be deduced that the solvent played crucial roles in the fabrication of hierarchically porous MOF. CuBTC-Water synthesized from Cu_2_O and water–ethanol solution possessed abundant hierarchical structures and a BET surface area of 1697 m^2^ g^−1^, higher than CuBTC-DMF (1335 m^2^ g^−1^) synthesized from Cu_2_O and water–ethanol–DMF solution.

Owing to the use of insolvable Cu_2_O as the metal precursor in water–ethanol, the formed CuBTC-Water had abundant deficiencies in the crystalline framework. X-ray photoelectron spectroscopy (XPS) confirmed the large amount of Cu (I) in the framework, which might have contributed to the deficiencies. Introduction of DMF solution helped to form more integrated crystalline frameworks with fewer deficiencies, ascribed to the high affinity of DMF to the metal sites, which accorded with lower Cu (I) amounts in CuBTC-DMF, as seen in the XPS results (Figure 2c). Therefore, CuBTC-Water exhibited hierarchically porous structures and a large BET surface area by virtue of the deficiencies formed during synthesis via Cu_2_O and water–ethanol solution.

**Figure 3:**
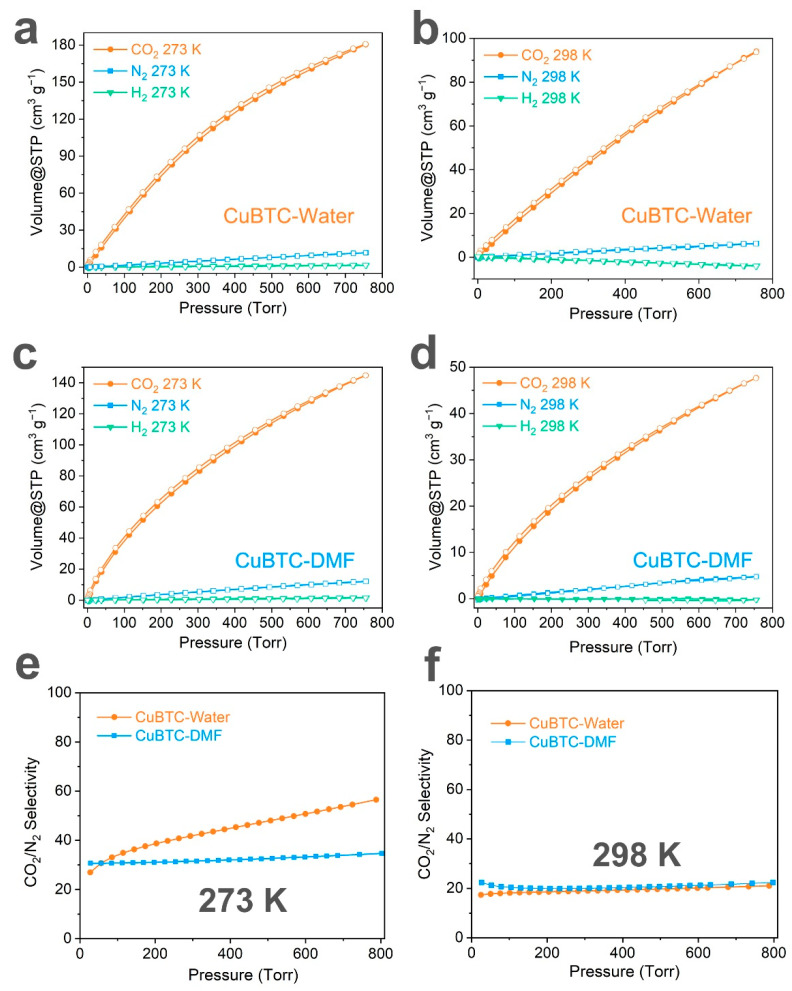
(**a**,**b**) Adsorption–desorption isotherms of CuBTC-Water at 273 K and 298 K. (**c**,**d**) Adsorption–desorption isotherms of CuBTC-DMF at 273 K and 298 K. (**e**,**f**) Selectivity of CuBTC-Water and CuBTC-DMF for CO_2_/N_2_ mixtures (CO_2_/N_2_ = 15:85 *v*/*v*, calculated using the IAST method) at 273 K and 298 K.

In the hierarchically porous structures of CuBTC-Water, mesopores are beneficial to facilitating the mass diffusion/transfer processes, and micropores contribute to the high storage ability of gas molecules. To investigate the CO_2_ adsorption properties, static equilibrium adsorption was carried out. The adsorption–desorption isotherms measured at 273 K and 298 K for CuBTC-Water are displayed in Figure 3a,b. CuBTC-Water demonstrated a CO_2_ uptake of 180.529 cm^3^ g^−1^ at 273 K and 1 bar and 94.147 cm^3^ g^−1^ at 298 K and 1 bar, with selectivity for CO_2_/N_2_ mixtures (CO_2_/N_2_ = 15:85 *v*/*v*) as high as 56.547 at 273 K (Figure 3e, calculated using the IAST method), whereas microporous CuBTC-DMF only exhibited 144.681 cm^3^ g^−1^ at 273 K and 1 bar (Figure 3c) and 47.70 cm^3^ g^−1^ at 298 K and 1 bar (Figure 3d) with a selectivity of 34.692 for CO_2_/N_2_ mixtures (Figure 3f). Owing to the higher BET surface areas and pore volume (0.757 cm^3^ g^−1^ of CuBTC-Water (micropore volume of 0.668 cm^3^ g^−1^) and 0.509 cm^3^ g^−1^ of CuBTC-DMF (micropore volume of 0.498 cm^3^ g^−1^), the formed hierarchically porous CuBTC-Water presented a higher CO_2_ adsorption ability and selectivity toward N_2_ and H_2_ than microporous CuBTC-DMF under static equilibrium adsorption.

Furthermore, according to the CO_2_/N_2_ adsorption and desorption isothermal data at 298 K and 273 K, we also calculated the CO_2_/N_2_ adsorption enthalpies of CuBTC-Water and CuBTC-DMF. As validated by Figure S7, the CO_2_/N_2_ adsorption enthalpy of CuBTC-Water material was obviously lower than that of CuBTC-DMF material. The adsorption enthalpy differences indicate that CuBTC-Water material not only possesses higher CO_2_ adsorption capacity, but also has lower CO_2_/N_2_ adsorption enthalpy, suggesting a much easier desorption barrier with low energy consumption for regeneration.

**Figure 4:**
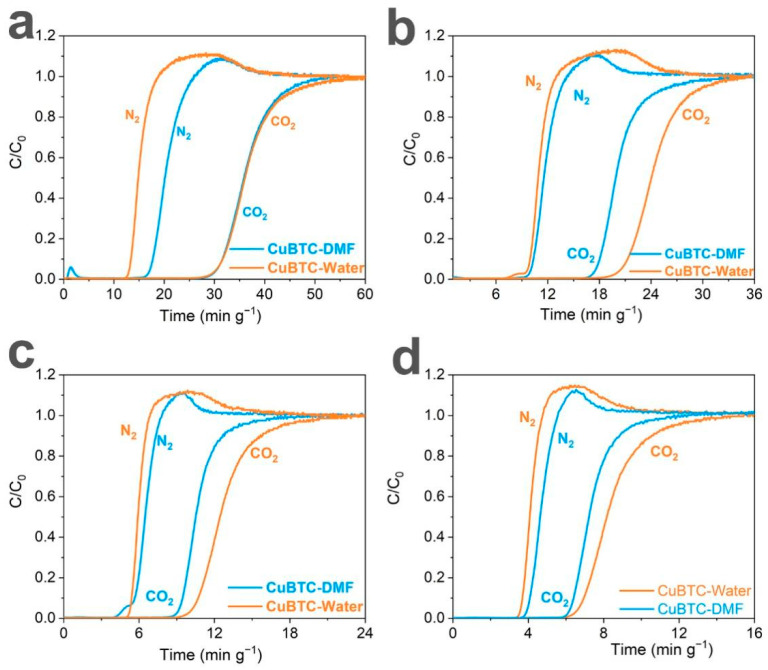
The dynamic breakthrough curves of CuBTC-DMF and CuBTC-Water for the CO_2_/N_2_ mixture (The feed gas for the breakthrough measurement and chromatographic separation was composed of 20% CO_2_, 20% N_2_ and 60% He.) at room temperature with different mass flow rates of (**a**) 10 sccm, (**b**) 20 sccm, (**c**) 50 sccm and (**d**) 100 sccm.

Moreover, in practical operations, gas separations are always conducted under a dynamic gas flow condition, in which the diffusions of gases within the adsorbents can impede the overall performance [14]. Hence, we conducted breakthrough measurements to test the separation ability of these two adsorbents under a dynamic flow condition. As displayed in Figure 4a–d, the breakthrough measurements for CuBTC-Water and CuBTC-DMF were conducted using a CO_2_/N_2_ gas mixture (20/20, *v*/*v*) under various gas flow rates at 298 K and 1 bar. The results show that CuBTC-Water demonstrated a much earlier breakthrough for N_2_ and a similar CO_2_ breakthrough time compared to CuBTC-DMF at the slow gas flow rate of 10 sccm (Figure 4a). At different gas flow rates, the hierarchically porous CuBTC-Water displayed an earlier nitrogen gas breakthrough and a greater amount of carbon dioxide adsorption than CuBTC-DMF.

**Figure 5:**
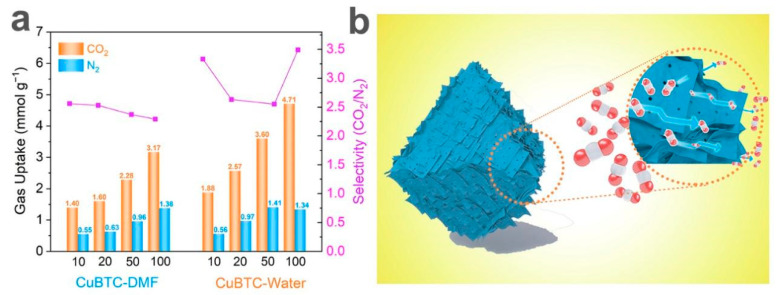
(**a**) Gas uptake amount and selectivity of CuBTC-DMF and CuBTC-Water. (**b**) Schematic illustration for CO_2_ separation under dynamic conditions through hierarchically porous CuBTC-Water. The circle area indicates the schematically enlarged MOF crystal for CO_2_/N_2_ separation.

The earlier N_2_ breakthrough indicated that N_2_ could be quickly saturated in the adsorbents and easily pass through the adsorbents with low diffusion resistance, which could be attributed to the mesoporous structures of CuBTC-Water with more exposed adsorptive surface, shortened gas diffusion length and low adsorption enthalpy. Thus, the CO_2_ adsorption amount of CuBTC-Water could reach 1.88 mmol g^−1^, higher than that of CuBTC-DMF. Thus, hierarchically porous CuBTC-Water demonstrated a selectivity of 3.38 at a gas flow rate of 10 sccm, which was much higher than that of microporous CuBTC-DMF, which demonstrated a selectivity of 2.56. Remarkably, as shown in Figure 5a, at a higher gas flow rate of 20, 50 and 100 sccm, CuBTC-Water also exhibited much better dynamic selectivity for CO_2_/N_2_ separation. CuBTC-Water exhibited an earlier N_2_ breakthrough and a much later CO_2_ breakthrough than CuBTC-DMF at the increased gas flow rates, indicating a better separation ability and the potential of hierarchically porous CuBTC-Water to achieve realistic CO_2_ separation. This further proved the superiorities of the hierarchically porous structures of CuBTC-Water, which can fully expose the adsorption surface area and facilitate gas diffusion under dynamic separation conditions (Figure 5b).

Compared with other reported synthesized compounds as listed in Table 1 and Table S1, the advantages of CuBTC-Water for efficient dynamic CO_2_ separation may be attributed to the following factors: firstly, this one-pot synthesis method with green environmental solvent is a quite simple preparation process compared to conventional methods, which is beneficial for its large-scale production and application. Secondly, well-organized hierarchical structures offer channels and shortened pathways, promoting realistic dynamic CO_2_ separation. Thirdly, the synergistic effect of hierarchical pores and moderate adsorption enthalpy also contribute to enhancing the separation efficiency.

## 4. Summary

In summary, the hierarchically porous CuBTC-Water was facilely synthesized through a self-sacrificed template method at room temperature utilizing Cu_2_O as the sacrificed template. In contrast to traditional methods of fabricating hierarchically porous MOFs, this work presents a one-pot route to constructing the hierarchical structures with abundant micropores and mesopores without any extra templates and post-treatments. Through simple tuning solvents with/without DMF, the porous structures can be intriguingly regulated from microporous structures to hierarchically porous structures. The hierarchically porous structures play vital roles in enhancing the separation ability of the CO_2_ gas mixture by virtue of the highly exposed adsorptive surface and shortened diffusion length. Hence, the produced hierarchically porous CuBTC-Water not only exhibited a high CO_2_ adsorption ability under static adsorption–desorption conditions, but also showed much higher selectivity for the CO_2_/N_2_ mixture under dynamic flow conditions than for CuBTC-DMF. The facile synthesis of hierarchical CuBTC-Water and its high adsorption/separation ability under dynamic flow conditions suggest the high potential of CuBTC-Water for realistic CO_2_ separation.

## Figures and Tables

**Table 1:** Comparison of the performance of CuBTC-Water with other relevant materials reported in the literature.

Materials	S_BET_ (m^2^ g^−1^)	CO_2_ Uptake	Ref.
CuBTC-Water	1697	94.147	This work
UiO-66-DAM	860	54.1	[29]
Zn porous MOF	447.92	59.2	[30]
NJFU-5	473	37.0	[31]
Co_3_(L)_2_ (2,2′-bipy)_2_](DMF)_3_(H_2_O)_3_	658	50	[32]
{[Co_2_(4,4′-bpy)(L)]·H_2_O·0.5(DMF)}_n_	224	36.4	[33]
TIBM-Cu MOF	1073.5	88.2	[34]
Qc-5-Cu	211.3	60.76	[35]

## Data Availability

The data presented in this study are available upon request from the corresponding author.

## Supplementary Materials

The following are available online at https://www.mdpi.com/article/10.3390/ma15155292/s1, Figure S1: The illustrated description for the breakthrough instruments. Figure S2: Enlarged the PXRD patterns of simulated CuBTC for 6 times in 2Ɵ > 13˚ range. Figure S3: XRD of CuBTC-Water after water stability test. Figure S4: BET fitting plot of CuBTC-DMF. Figure S5: BET fitting plot of CuBTC-Water. Figure S6: Enlarged pore distributions of CuBTC-DMF and CuBTC-Water calculated by using NLDFT method. Figure S7: Adsorption enthalpies of (a) CO_2_ and (b) N_2_ for CuBTC-DMF and CuBTC-Water. Table S1: Comparison of the other reported numbers for CuBTC in literature [36,37,38,39,40].
