# Supplementary material for: Template-Mediated Synthesis of Hierarchically Porous Metal–Organic Frameworks for Efficient CO2/N2 Separation
Source: Materials (Basel). 2022 Jul 31;15(15):5292. doi: 10.3390/ma15155292 (PMC9369960; doi:10.3390/ma15155292)
Supplement: Supplementary file 1 [file materials-15-05292-s001.zip › materials-1790247-supplementary.pdf]

Template-Mediated Synthesis of Hierarchically Porous Metal–Organic Frameworks for Efficient CO_2/N_2 Separation

Tianjie Qiu[1], Song Gao[1,*], Yanchun Fu[2], Dong Xu[2], and Dekai Kong[3]

[1] *Beijing Key Laboratory for Theory and Technology of Advanced Battery Materials, School of Materials Science and Engineering, Peking University, Beijing 100871, China*

[2] *CHN Energy New Energy Technology Research Institute Co., Ltd., Beijing 102211, China*

[3] *State Key Laboratory of Heavy Oil Processing, China University of Petroleum-Beijing, Beijing 102249, China*

* Correspondence: gaosong198600@pku.edu.cn (Song Gao)

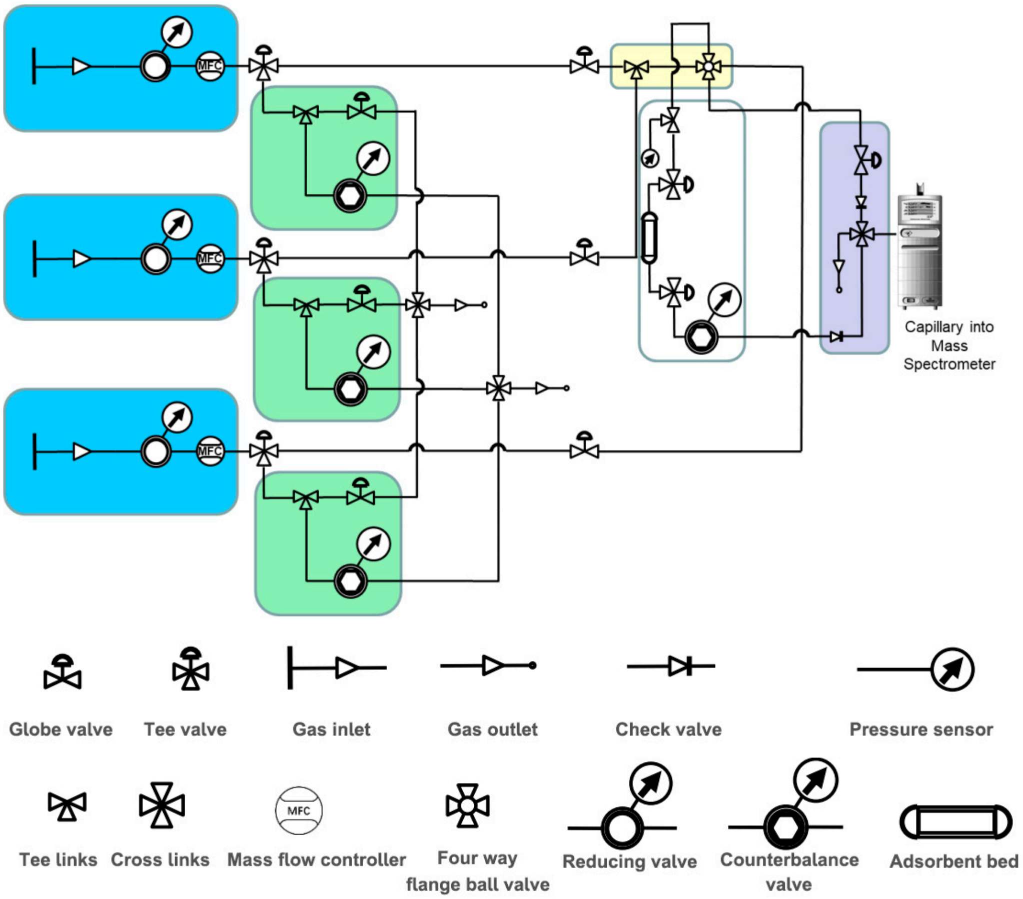

Figure S1. The illustrated description for the breakthrough instruments.

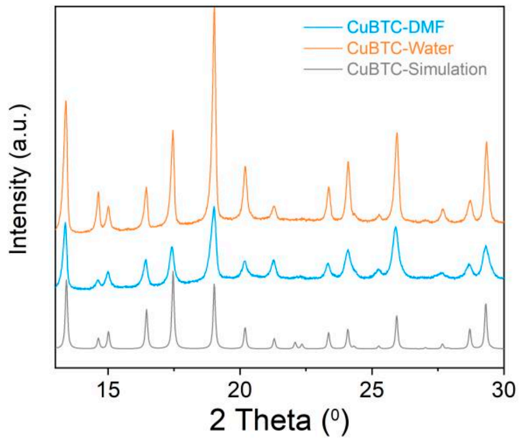

Figure S2. Enlarged the PXRD patterns of simulated CuBTC for 6 times in 2ϴ > 13˚ range.

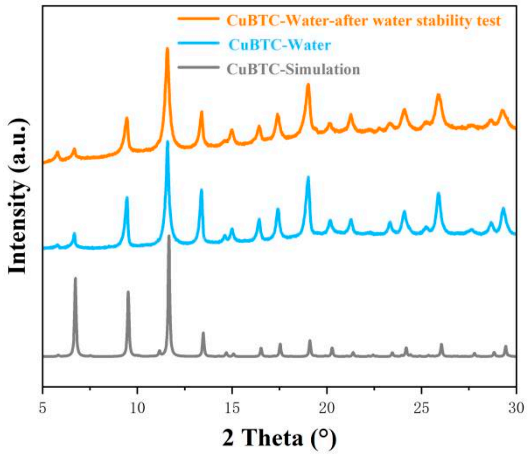

Figure S3. XRD of CuBTC-Water after water stability test.

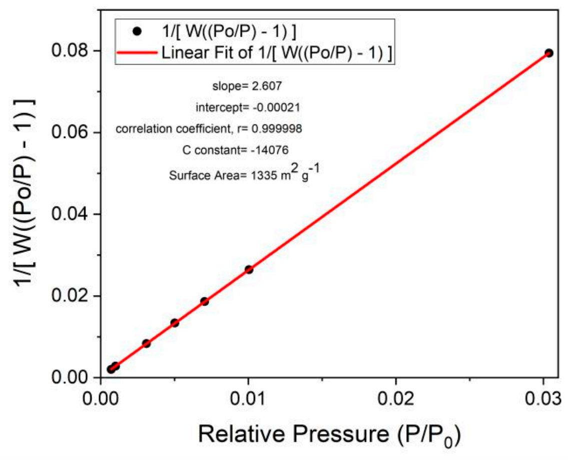

Figure S4. BET fitting plot of CuBTC-DMF.

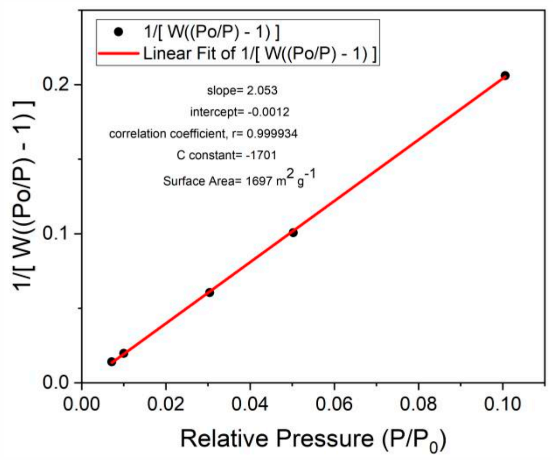

Figure S5. BET fitting plot of CuBTC-Water.

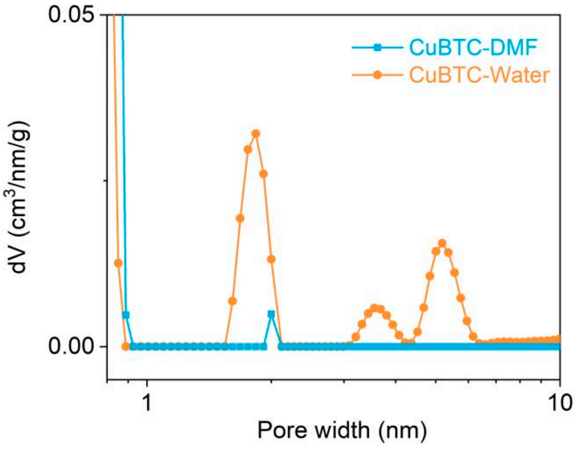

Figure S6. Enlarged pore distributions of CuBTC-DMF and CuBTC-Water calculated by using NLDFT method.

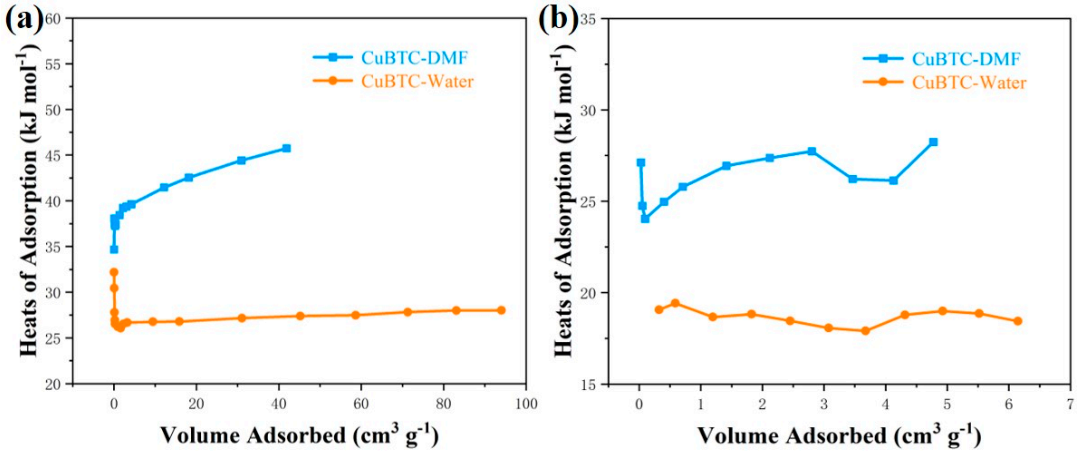

Figure S7. Adsorption enthalpies of (a) CO_2 and (b) N_2 for CuBTC-DMF and CuBTC-Water

Table S1. Comparison of the other reported numbers for CuBTC in literature.

materials	S_{BET} ($m^2\ g^{-1}$)	Ref.
CuBTC-Water	1697	This work
CuBTC-DMF	1335	This work
$[Cu_3(TMA)_2(H_2O)_3]_n$	692.2	[1]
HKUST-1	1346	[2]
$Cu_3(BTC)_2$	1529	[3]
ED@$Cu_3(BTC)_2$-1	444	[3]
ED@$Cu_3(BTC)_2$-2	163	[3]
H-HKUST-1_A1	1209	[4]
H-HKUST-1_B1	1216	[4]
H-HKUST-1_C1	1354	[4]
H-HKUST-1[f]	1763	[4]
HKUST-1[g]	1895	[4]
3D-nets	680	[5]
Octahedrons	1060	[5]
HKUST-1 etching in pH 2.6 for 240h	1117	[6]

1. Chui, S. S. Y.; Lo, S. M. F.; Charmant, J. P. H.; Orpen, A. G.; Williams, I. D., *Science*, **1999**, *283*, 1148-1150.
2. Huo, J.; Brightwell M.; Hankari, S. E.; Garai, A.; Bradshaw, D., A versatile, industrially relevant, aqueous room temperature synthesis of HKUST-1 with high space-time yield. *J. Mater. Chem. A*, **2013**, *1*, 15220-15223.
3. Zhong, R. Q.; Yu X. F.; Meng, W.; Han, S. B.; Liu, J.; Ye, Y. X.; Sun, C. Y.; Chen, G. J.; Zou, R. Q., A solvent 'squeezing' strategy to graft ethylenediamine on $Cu_3(BTC)_2$ for highly effificient CO_2/CO separation, *Chem. Eng. Sci.*, **2018**, *184*, 85-92.
4. Duan, C. X.; Zhang, H.; Li, F. E.; Xiao, J.; Luo, S. J.; Xi, H. X., Hierarchically porous metal-organic frameworks: rapid synthesis and enhanced gas storage, *Soft Matter*, **2018**, *14*, 9589-9598.
5. Zhan, G. W.; Zeng, H. C., An alternative synthetic approach for macro-meso-microporous metal-organic frameworks via a "domain growth" mechanism, *Chem. Commun.*, **2016**, *52*, 8432-8435.
6. Doan, H. V; Sartbaeva, A.; Eioi, J.-C.; Davis, S. A.; Ting, V. P., Defective hierarchical porous copper-based metal-organic frameworks synthesised via facile acid etching strategy, *Sci. Rep.*, **2019**, *9*, 10887.
